# TPM3-NTRK1 Fusion Cervical Sarcoma: A Case Report of a Novel Subset of Gynaecological Sarcomas and Successful Treatment of Recurrent Disease With Trk-Inhibition Therapy

**DOI:** 10.7759/cureus.95363

**Published:** 2025-10-25

**Authors:** Jack Lowe-Zinola, Mirna Elghobashy, Matthew Evans, Talha Hafeez, Alaa El-Ghobashy

**Affiliations:** 1 Gynaecological Oncology, Royal Wolverhampton NHS Trust, Wolverhampton, GBR; 2 Obstetrics and Gynaecology, Royal Wolverhampton NHS Trust, Wolverhampton, GBR; 3 Pathology and Laboratory Medicine, Royal Wolverhampton NHS Trust, Wolverhampton, GBR; 4 Oncology, Royal Wolverhampton NHS Trust, Wolverhampton, GBR; 5 Gynaecological Oncology Division, American Hospital Dubai, Dubai, ARE

**Keywords:** cervix, gynaecological, neurotrophic tyrosine kinase receptor (ntrk), recurrent disease, sarcoma

## Abstract

Neurotrophic tyrosine kinase receptor (*NTRK*) activation by neurotrophins has been shown to promote cellular proliferation, differentiation, and survival by preventing apoptosis. *NTRK* gene fusions are rare in gynaecological cancer. This case report describes the successful management of a metastatic *NTRK*-positive cervical sarcoma using Trk-inhibition therapy, following recurrence after surgical interventions and chemotherapy. The patient was administered 31 cycles of 100 mg larotrectinib (Vitrakvi) twice daily. The patient remains stable with no evidence of local, nodal, or metastatic disease for more than three years following the initial presentation. This report aims to propose the use of larotrectinib as a management option for recurrent *NTRK*-positive cervical sarcomas.

## Introduction

Neurotrophic tyrosine kinase (*NTRK*) 1, 2, and 3 genes encode for tropomyosin receptor kinases (Trk) A, B, and C, respectively. These receptors are primarily expressed in neuronal tissue, and their activation by neurotrophins has been shown to promote cellular proliferation, differentiation, and survival by preventing apoptosis [1]. The *NTRK* gene alteration can lead to carcinogenesis in both neurogenic and non-neurogenic tissues. There are various alterations, including mutation, deletion, and fusion. Fusions of the *NTRK* genes were first demonstrated in a colorectal tumour in 1986 [2], and, more recently, *NTRK* fusions have been identified in many cancers of varying age groups, including adult and paediatric solid tumours [3-8]. *NTRK* fusions have also been described in mesenchymal tumours of soft tissue origins.

The detection of *NTRK* fusions is now of clinical significance after the development and approval of Trk-inhibitor therapy. Furthermore, it has been shown that *NTRK* fusion may be the only pathological genomic rearrangement in some tumours and, as such, may be the only target for specific therapy [9]. In April 2020, the National Institute for Health and Care Excellence (NICE) approved larotrectinib (Vitrakvi), a drug that inhibits all of Trk-A, B, and C, for locally advanced or metastatic *NTRK* fusion-positive solid tumours where no other satisfactory treatment option exists, regardless of histological subtype [10]. Clinical trials examining the efficacy of larotrectinib for *NTRK* fusion-positive tumours in adult and paediatric patients have demonstrated an overall response rate of 75%, with 55% of patients remaining progression-free, and with acceptable side effect rates [11]. In this paper, we present a case of recurrent metastatic *NTRK*-positive cervical sarcoma, which was successfully treated with Vitrakvi. She remains alive with no evidence of disease 36 months following initial presentation.

## Case presentation

**Figure 1:**
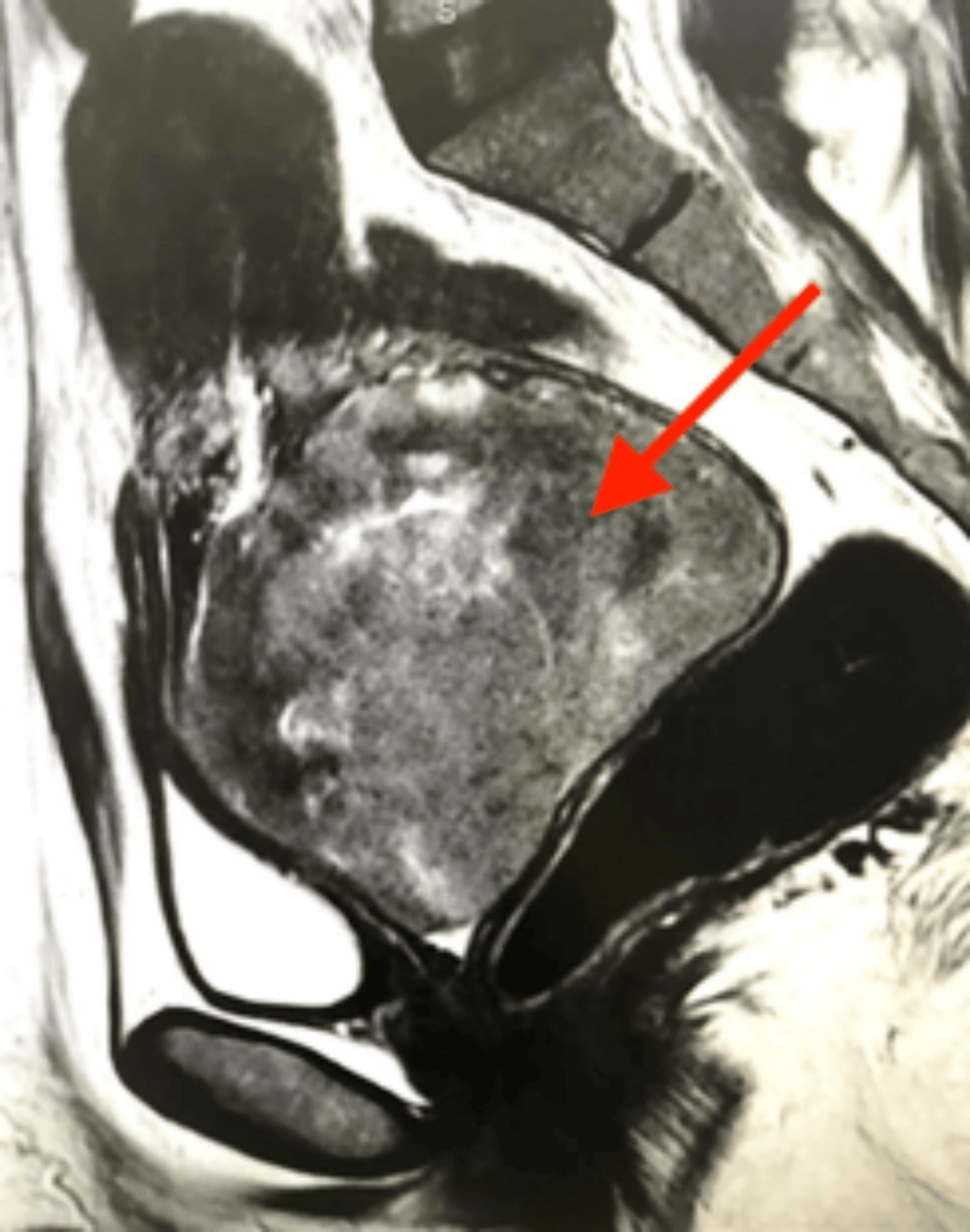
Sagittal section of T2-weighted MRI of the pelvis showing an exophytic tumour mass arising from the posterior cervix (red arrow) and filling the upper vagina measuring 9.3 cm.

A 49-year-old woman was referred to the gynaecology clinic with heavy vaginal bleeding with blood clots. The patient was very pale and lethargic. She was known for hypothyroidism and was on levothyroxine tablets. Vaginal examination confirmed offensive bloody vaginal discharge and a large mass of 10 cm filling the upper half of the vagina. It was not possible to determine whether the mass was originating from the cervix or had prolapsed from the uterus. An outpatient biopsy revealed a high-grade invasive spindle cell tumour. A subsequent magnetic resonance imaging (MRI) of the abdomen and pelvis (Figure 1) showed a 10 cm x 9 cm cervical tumour with a suspicion of very early parametrial invasion. The uterus was small in size (8 cms) with no endometrial or myometrial abnormalities. There was no evidence of pelvic or para-aortic lymph node enlargement. The abdominal solid organs were normal, and there was no ascites. The chest computed tomography (CT) scan thorax was clear of lung and other distant metastases.

The case was discussed at the West Midlands regional sarcoma multidisciplinary meeting (MDM) when it was agreed to proceed with primary radical surgery. The patient underwent midline laparotomy, antegrade total abdominal hysterectomy, and bilateral salpingo-oophorectomy. No lymphadenectomies were attempted as there were no enlarged lymph nodes in all retroperitoneal spaces. Prior ureteric stenting was performed to facilitate the surgery, which was uneventful. The patient made an excellent recovery and was discharged home on day 4.

**Figure 2:**
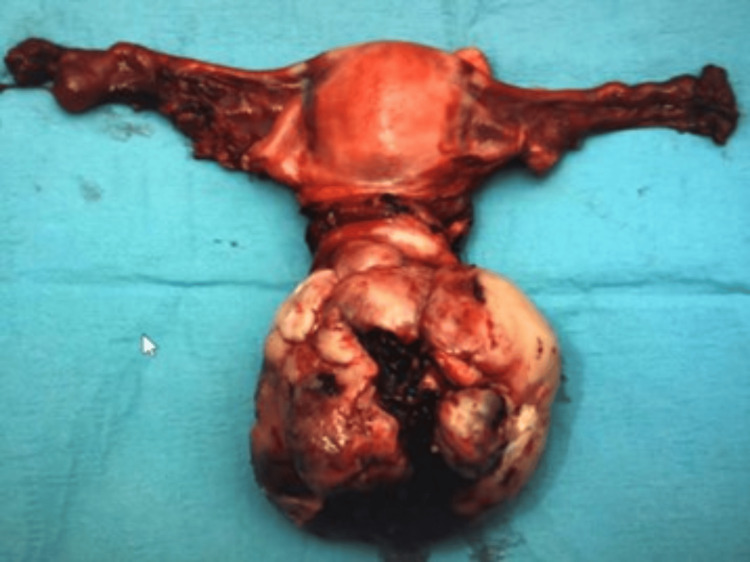
Surgically resected specimen of the uterus, cervix with large cervical tumour arising from the posterior lip, and both fallopian tubes.

**Figure 3:**
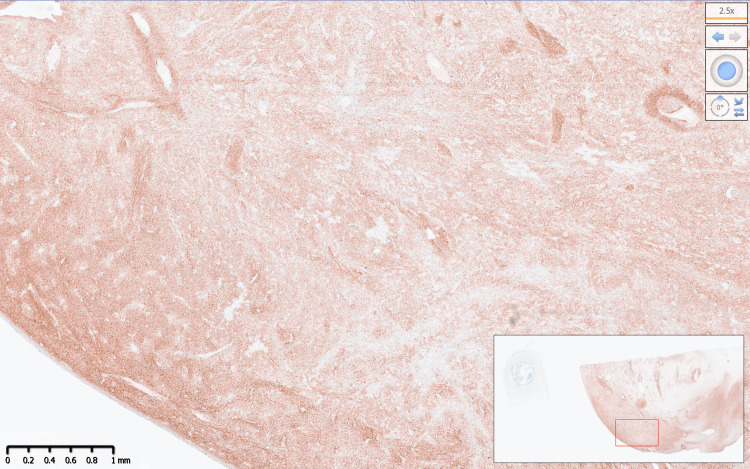
Immunohistochemical staining for pan-tyrosine receptor kinase detection (VENTANA pan-TRK (EPR17341) assay) at 2.5x magnification.

**Figure 4:**
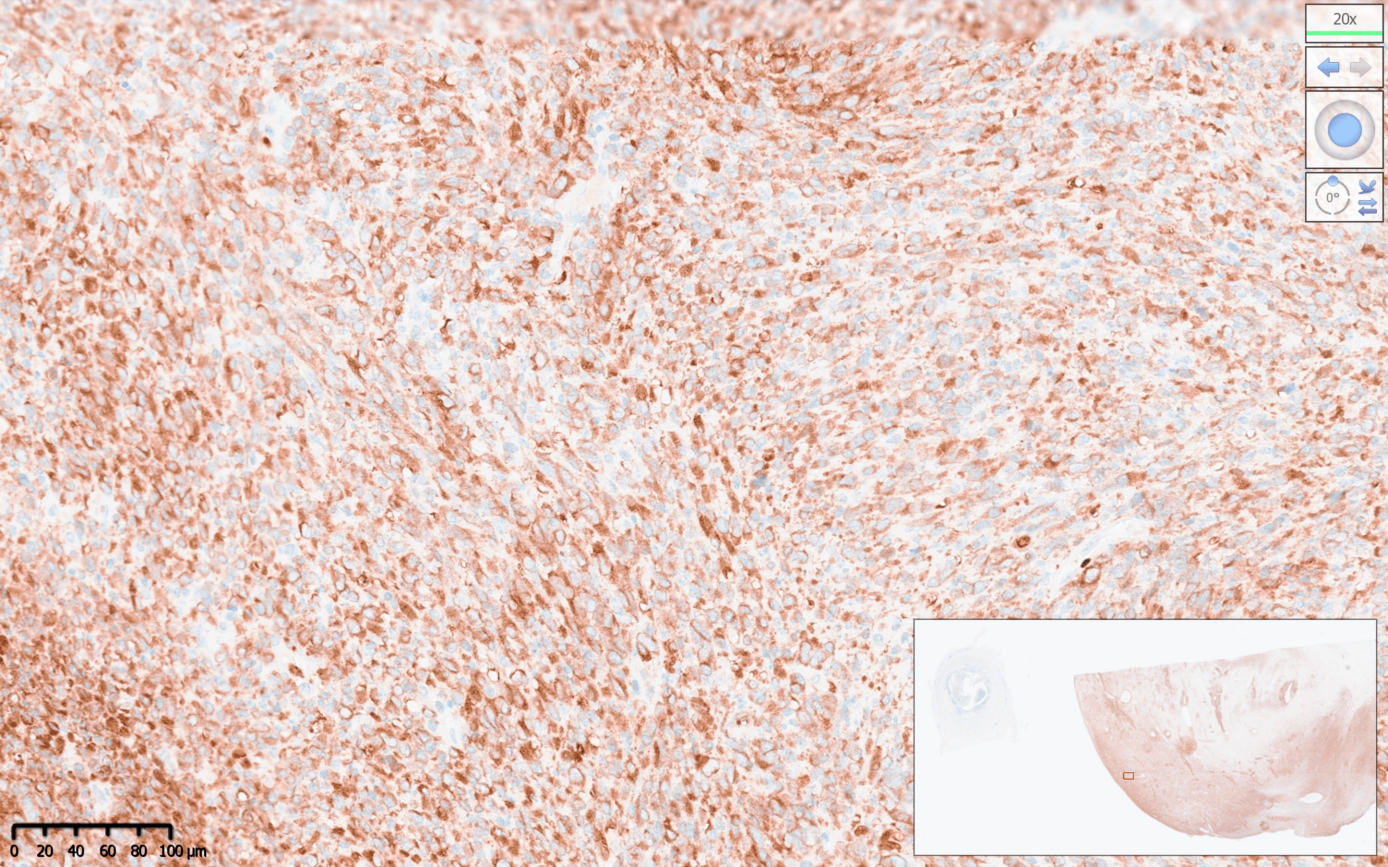
Immunohistochemical staining for pan-tyrosine receptor kinase detection (VENTANA pan-TRK (EPR17341) assay) at 20x magnification.

**Figure 5:**
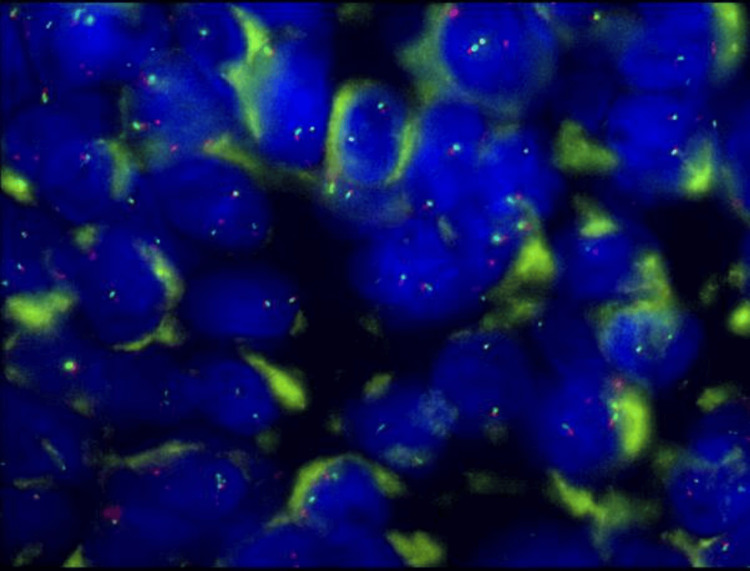
FISH testing (Zytovision ZytoLight SPEC NTRK1 Dual-Colour Break-Apart Probe) that confirmed the presence of an NTRK1 translocation with a proportion of cells showing separation of red and green signals >2 signal distances apart.

Macroscopically, the specimen weighed 420 grams and consisted of a uterus, cervix with an attached cervical tumour arising from the posterior lip of 10x10 cm, both fallopian tubes, and ovaries (Figure 2). The histology report confirmed a poorly differentiated malignant cervical tumour composed of predominantly spindle cells, with moderate to severe pleomorphism and high mitotic activity (>10 per high-power field). Immunohistochemistry showed that the tumour was diffusely positive for CD99; focally positive for cyclin-D1, CD10, CD34, BCL2; and negative for cytokeratins, S100, HMB45, SMA, desmin, caldesmon, estrogen receptor (ER), progesterone receptor (PR), neuroendocrine markers, and gastrointestinal stromal tumour (GIST) markers. Staining for p53 showed wild-type expression, and beta-catenin showed only cytoplasmic staining. Pan-tyrosine receptor kinase immunohistochemistry (VENTANA pan-TRK (EPR17341) Assay, Roche, Switzerland) was positive (Figures 3, 4). Subsequent fluorescence in situ hybridisation (FISH) testing (Zytovision ZytoLight SPEC *NTRK1* Dual Colour Break Apart Probe, ZytoVision GmbH, Germany) confirmed the presence of an *NTRK1* translocation, and the next-generation sequencing (Life Technologies Oncomine Focus Panel, ThermoFisher Scientific, USA) showed that this was a TPM3-*NTRK1* fusion (Figure 5).

Postoperative whole-body CT scan demonstrated no residual disease, and the tumour was staged as per the International Federation of Gynecology and Obstetrics (FIGO) system for leiomyosarcoma as stage 1B, as there was involvement of the posterior vaginal wall. The patient remained well with no clinical evidence of recurrent disease on three monthly surveillance visits. After a period of close observation for nine months, the patient presented with heavy vaginal bleeding. A 7 cm mass was seen on clinical examination arising from the vaginal vault. She was counselled for examination under anaesthetics and biopsy/resection of the mass. During surgery, the mass was 7 cm and attached with a narrow stalk to the vaginal vault. There was no surgical evidence of vaginal or parametrial invasions. The histology of the resected specimen later confirmed recurrent *NTRK*-positive sarcoma similar to the original diagnosis. There was a suspicion of pulmonary metastases on the postoperative CT scan chest. After discussion at the MDM, the patient received four cycles of doxorubicin (60 mg/m^2^). A follow-up CT scan after three months confirmed progressive disease with multiple pulmonary metastases. The patient was then deemed eligible for Trk-inhibition therapy with larotrectinib (Vitrakvi), and she received 31 cycles of 100 mg orally twice daily. Following this treatment, reductions in the size of the pulmonary metastases were observed, as well as no further progression of the pelvic mass. Under close follow-up surveillance for more than 36 months post initial presentation, the patient remains stable with no evidence of local recurrence or metastatic disease.

## Discussion

Increasing evidence suggests that *NTRK* fusion results in persistent elevation or activation of TRK proteins. Moreover, these TRK family members have been shown to stimulate cancer cell proliferation and trigger various downstream signalling pathways, which could contribute to tumorigenesis. These discoveries underscore the significance of *NTRK* fusion in the advancement of malignancy [12]. 

The initial identification of the *NTRK* gene fusion occurred in 1986 within a colorectal cancer patient [2]. Subsequently, this fusion has been documented in approximately 2% of individuals diagnosed with this form of cancer. Noteworthy traits among colorectal cancer patients harbouring *NTRK* gene fusions include female gender, right-sided colon location, and microsatellite instability phenotype [13].

The prevalence of *NTRK* gene fusions in paediatric cancers mirrors the broader pattern observed in adult cancers. Neuroblastoma, Wilms tumor, and hepatoblastoma exhibit low rates of *NTRK* gene fusions. Conversely, *NTRK* gene fusions are more frequently encountered in certain rare childhood cancers, including infantile fibrosarcomas, gliomas, and thyroid cancers. Those cancers harbouring *NTRK* gene fusions often exhibit favourable responses to TRK inhibitors, with reported objective response rates reaching up to 90% [14].

Sarcomas, a rarity among adult cancers at just 1% of cases, present a complex landscape with over 70 histologic subtypes. The challenge in treating sarcomas lies in their heterogeneity and the scarcity of actionable genetic abnormalities, coupled with their inherent resistance to standard chemotherapy. Within this context of limited treatment options, targeting *NTRK* gene fusion stands out as a promising approach in managing sarcoma patients. It may even serve as a frontline therapy in selected cases where other treatment avenues are lacking [15].

In this report, we present a case of TPM3-*NTRK1*-positive recurrent metastatic cervical sarcoma that was successfully treated with larotrectinib. We have shown that the patient sustained remission on Trk-inhibition therapy for a prolonged duration of more than 36 months. We were able to identify and confirm the genetic abnormality in this case using a combined methodology involving immunohistochemistry (IHC), fluorescent in situ hybridization (FISH), and next-generation sequencing (NGS). 

Identification of *NTRK* gene fusions in tumour tissue may be achieved using a number of assays, including IHC and next-generation NGS-based analyses. The diagnostic implications, advantages, and limitations of different testing methods have become better understood over time and have been discussed and described recently in the literature [16,17]. Ribonucleic acid (RNA) NGS panel testing can provide a high sensitivity and specificity in the detection of *NTRK* fusions and may be considered as the gold standard technique. The use of RNA avoids the detection of non-functional rearrangements and allows for the detection of rearrangements involving multiple other genes simultaneously. However, its limitations include a higher cost, and, although panels differ in their tolerance for low-quality RNA, a relatively high failure rate, and a requirement for high-quality RNA. Deoxyribonucleic acid (DNA) NGS panel testing can also be performed, although it is more likely to detect non-functional rearrangements that cannot be targeted. FISH may be an alternative method where NGS is not possible, for example, in cases where RNA is degraded. It does, however, run the risk of picking up non-functional rearrangements and requires three separate FISH tests to assess each of the three *NTRK* genes, which is time-consuming and results in a high cost. The use of IHC to detect *NTRK* fusions is a quicker and more readily available test in many laboratories and is associated with lower costs. However, positive IHC requires confirmation using another testing method, and there is no formal definition of immunohistochemical positivity. It is also now known that a subset of high-grade endometrial stromal sarcomas show convincing IHC positivity for *NTRK* fusions despite not harbouring rearrangements [13]. In addition, of concern is the finding of false negative results, particularly for *NTRK3* fusions, for which IHC has a lower sensitivity as compared with *NTRK1* and *NTRK2* rearrangements.

Few recent studies have demonstrated *NTRK* fusions in gynaecological sarcomas, which otherwise lacked diagnostic features attributable to defined mesenchymal tumours [9-11,18-20]. This has led to the description of a novel subset of tumours known as *NTRK*-rearranged spindle cell neoplasms. These tumours affect premenopausal women with a median age of 39 and are likely to morphologically resemble fibrosarcoma [17-20], although those that resemble adenosarcoma have also been described [18]. Various fusion partners have been described, including TPM3-*NTRK1*, LMNA-*NTRK1*, RBPMS-*NTRK3*, TPR-*NTRK1*, EML4-*NTRK3*, and SPECC1L-*NTRK3* [14,16,17,18], with TPM3-*NTRK1* being the most common and present in more than 60% of these tumours [18]. These tumours are likely to be aggressive; Chiang et al. described four patients with *NTRK* fusion-positive gynaecological sarcomas all diagnosed at FIGO stage 1B (as per the leiomyosarcoma staging system), with two recurring seven and 12 months following presentation, despite optimal surgical resection, and with the latter patient ultimately dying from the disease 78 months after initial presentation despite adjuvant chemotherapy [18]. In our case, the recurrence of sarcoma was detected nine months following the initial complete resection. Other reported cases have demonstrated a clinically disease-free state at the 11-month follow-up with a combination of primary surgery and adjuvant pelvic radiotherapy and brachytherapy [19]. Supporting the evidence outlined in our case regarding the effectiveness of targeted Trk-inhibition therapy, another case reported in the literature describes a patient with *NTRK* fusion-positive cervical sarcoma. Initially treated with primary surgery, this patient developed pleural metastasis during the 16-month follow-up period. Subsequently, they initiated Trk-inhibitor therapy with larotrectinib and remained disease-free for an additional 15 months [20]. Our case with lung metastasis, however, is still alive with no evidence of radiological progression more than 36 months following the initial presentation.

## Conclusions

*NTRK* gene fusions are important oncogenic events in various tumour types. They appear to define a novel subset of gynaecological sarcomas with morphological features distinct from other gynaecological mesenchymal tumours. Targeted therapy with Trk inhibition has been shown to produce positive response rates across adult and paediatric *NTRK* fusion-positive tumours, and there are reports of efficacy in *NTRK* fusion gynaecological sarcomas. Our case corroborates those reported in the literature. We have demonstrated excellent durable response and improved survival for our patient with this treatment. Larotrectinib has been approved by the NICE for use in locally advanced or metastatic *NTRK* fusion-positive tumours. Patients who may benefit from Trk inhibition can be referred for this targeted therapy. Testing for *NTRK* fusions should be considered for gynaecological sarcomas that are not readily classifiable to avoid denying patients this potentially beneficial treatment modality.

With increasing numbers of patients and outcome data over longer periods of time, cost-effectiveness and quality-adjusted life-year analyses will be required to examine the efficacy of this therapy against currently established treatment pathways. Considering that these novel *NTRK* fusion gynaecological sarcomas affect pre-menopausal women, future research might examine Trk inhibition as a neoadjuvant therapy for women desiring fertility-sparing cancer treatment. Further studies will also be required to understand their clinical behaviour as compared with more common gynaecological sarcomas.
